# Performance and Structure Evaluation of Gln-Lys Isopeptide Bond Crosslinked USYK-SPI Bioplastic Film Derived from Discarded Yak Hair

**DOI:** 10.3390/polym14122471

**Published:** 2022-06-17

**Authors:** Ruirui Wang

**Affiliations:** Department of Applied Chemistry, College of Chemistry and Chemical Engineering, Qinghai Normal University, 38 Wusi West Road, Xining 810008, China; wangruirui34@qhnu.edu.cn; Tel.: +86-13997280624

**Keywords:** yak keratin, soy protein isolate, bioplastic film, Gln-Lys isopeptide bonds, yak hair

## Abstract

To reduce the waste from yak hair and introduce resource recycling into the yak-related industry, an eco-friendly yak keratin-based bioplastic film was developed. We employed yak keratin (USYK) from yak hair, soy protein isolate (SPI) from soybean meal as a film-forming agent, transglutaminase (EC 2.3.2.13, TGase) as a catalytic crosslinker, and glycerol as a plasticizer for USYK-SPI bioplastic film production. The structures of the USYK-SPI bioplastic film were characterized by scanning electron microscopy (SEM), differential scanning calorimetry (DSC), and X-Ray diffraction (XRD). The mechanical properties, the thermal behavior, light transmittance performance, and water vapor permeability (WVP) were measured. The results revealed that the added SPI possibly acted as a reinforcement. The formation of Gln-Lys isopeptide bonds and hydrophobic interactions led to a stable crosslinking structure of USYK-SPI bioplastic film. The thermal and the mechanical behaviors of the USYK-SPI bioplastic film were improved. The enhanced dispersion and formation of co-continuous protein matrices possibly produced denser networks that limited the diffusion of water vapor and volatile compounds in the USYK-SPI bioplastic films. Moreover, the introduction of SPI prompted the relocation of hydrophobic groups on USYK molecules, which gave the USYK-SPI bioplastic film stronger surface hydrophobicity. The SPI and USYK molecules possess aromatic amino residuals (tyrosine, phenylalanine, tryptophan), which can absorb ultraviolet radiation. Thus, the USYK-SPI bioplastic films were shown to have an excellent UV barrier. The synergy effect between USYK and SPI is not only able to improve rigidity and the application performance of keratin-based composite film but can also reduce the cost of the keratin-based composite film through the low-cost of the SPI alternative which partially replaces the high-cost of keratin. The data obtained from this research can provide basic information for further research and practical applications of USYK-SPI bioplastic films. There is an increasing demand for the novel USYK-SPI bioplastic film in exploit packaging material, biomedical materials, eco-friendly wearable electronics, and humidity sensors.

## 1. Introduction

We are currently experiencing an era of overdependence on fossil fuels and increasingly serious environmental pollution. Determining how can we better manage the world’s waste is an urgent problem that needs to be solved. Compared with synthetic chemicals, natural proteins offer several advantages, such as being abundant, cheap, renewable, and biodegradable. Natural proteins are considered promising resources for the development of bio-based films as alternatives to plastic films synthesized from petroleum [1,2,3]. At present, there are two types of proteins (animal proteins and plant proteins) that can be used to prepare environmentally friendly bioplastic films. Among the animal proteins are keratin [4], collagen [5], and casein [6], and so on. Plant proteins mainly include soy protein isolate (SPI) [7], zein [8], wheat protein [9], and so on. The development of natural multifunctional protein film can effectively reduce the use of petroleum-derived polymers and thus reduce environmental pollution, which is in line with the sustainable development strategy. In particular, proteins extracted from discarded animal and plant tissues can be converted intomultifunctional and degradable protein film, which can ensure waste transformation in high-value products as well as energy conversation and emission reduction.

As an iconic symbol of the Qinghai-Tibetan Plateau, the yak is a necessary material for life and production for herdsmen living in high altitude areas [10]. Since the yak is a unique type of livestock that lives in the high-altitude, unpolluted, and harsh environments in the Qinghai-Tibet Plateau, it is regarded as an unpolluted animal [11]. Yaks contain the highest quality proteins, such as yak collagen, yak keratin (USYK), and yak casein. Every year, more than 40,000 tons of yak hair are produced in China [12]. Similar to wool, yak hair is widely used in textiles because of its excellent thermal insulation properties [13]. However, in China, a country where large scale channel yak farming occurs, a significant quantity of short, and crude yak hair that cannot be spun is discarded during textile, tanning, and food processing, resulting in environmental pollution [14]. Determining how to transform this discarded yak hair into products with high added value is key to the development of yak-related industry.

Keratin is a common biological polymer that is found in mammalian, avian, and reptilian epidermal appendages such as hair, nails, feathers, beaks, horns, hooves, whale baleen, scales, and other dead tissues [15,16]. Being a sulfur-rich structural protein with good cytocompatibility, keratin is an interesting candidate for the development of eco-friendly, multifunctional, and biodegradable bioplastic films. The amino acids cysteine, glutamate, aspartate, threonine, leucine, proline, serine, and glycine are abundant in keratin, giving keratin film exceptional functional qualities [17,18,19,20]. To extract keratin from natural hair, it is necessary to destroy the inter- and intra-molecular activity of natural hair by breaking disulfide bonds, hydrogen bonds, ionic bonds, van der Waals forces, and so on. Reduction, oxidation, ionic liquids, enzymatic lysis, and microwave-assisted extraction are usually used to destroy these interactions [21,22,23,24]. The mechanical and thermal properties of pure keratin film are poor, because the natural structure of keratin is destroyed during the extraction process to a certain extent. The application scope of pure keratin film is greatly limited. The composite modification approach is an effective, low-cost, and easy operation used to develop keratin-based functional film [14]. It is a desirable strategy to promote the structural and functional properties of synthetic biopolymers.

SPI is a plant protein derived from soybean meals with high commercial value. It is a safe raw material (GRAS) for preparing protein film, because it comes from plant tissue, which eliminates the risk of transmitting diseases. SPI consists of globulin (11S) and β-Paraglobulin (7S). SPI is widely used in the preparation of packaging films. It is possible to modify certain functional groups in keratin and SPI, including carboxylic, amino, and sulfhydryl groups, during composite film preparation [25]. Using the differences in molecular structure and properties between keratin and soybean proteins, a network structure including keratin and SPI can be produced through non-covalent bonds such as hydrogen bonds and Van der Waals forces. This can effectively improve rigidity and application performance of keratin-based composite film. At the same time, the cost of keratin-based composite film can be reduced by partially substituting keratin with low-cost of SPI.

Various chemicals have been employed as crosslinkers to fabricate the keratin-based composite films [26]. These chemical crosslinkers can form intra- and inter-molecular covalent bonds and can tailor the structure properties of macromolecules in keratin-based biopolymers. In this way, keratin-based composite film has a dense network structure, resulting in significantly improved functional properties in the films [6]. Traditional chemical crosslinkers include aldehydes, carbodiimides, epoxy compounds, and acylazides [27]. The chemical crosslinkers can significantly enhance the mechanical strength and reduce the antigenicity of the protein film. However, the obtained crosslinked materials are highly cytotoxic, limiting their application [28]. In terms of non-toxic, feasible, and efficient attributes, the biological enzymatic catalytic crosslinking is considered to be an ideal candidate for improving the physical properties of protein film [29]. Transglutaminase (EC 2.3.2.13, TGase) from microorganisms has been widely investigated for the preparation of protein film. The catalysis of TGase creates bonds between the γ-hydroxyamide group of glutamine residues and ε-Amino of Lysine residues, forming ε-(γ-Glutamyl) lysine Gln-Lys isopeptide bonds [30]. It is possible to further enhance the network structure of protein film by coupling an abundant number of Gln-Lys isopeptide bonds, thus endowing the protein film with ideal properties. Based on this, we decided to choose the TGase catalytic crosslinking approach to process the USYK-SPI bioplastic film in this study.

In our previous research [31], yak keratin (USYK) was successfully extracted from discarded yak hairs by the urea and sodium sulfide combined method. In the present study, in order to make good use of this discarded yak hair and reduce environmental pollution, a novel USYK-SPI bioplastic film was prepared by Gln-Lys isopeptide bond crosslinking. It is reasonable to believe that the use of plant protein (SPI) to partially replace animal protein (USYK) during film formation produces a certain synergistic effect. It can not only optimize the structure of the USYK-SPI bioplastic film and endow the USYK-SPI bioplastic film with unique functions but can also reduce the production cost and expand the application range of bioplastic film.

This study attempted to obtain keratin based composite film with a superior performance. The effects of SPI on the performance of the USYK-SPI bioplastic films were systematically investigated. The Gln-Lys isopeptide bond crosslinked USYK-SPI complexes were hypothesized to modify the microstructure and application performances of the USYK-SPI bioplastic films. The results of this study provide new ideas for the production of new keratin-based multifunctional film.

## 2. Materials and Methods

### 2.1. Materials and Chemicals

Yak hair was obtained from a woolen mill in Xining, (Qinghai, China). Soy protein isolate was bought from Shyuanye Biological Science and Technology Co., Ltd. (Shanghai, China). Transglutaminase (TGase, EC 2.3.2.13, 100 U/g proteins) was bought from Yiming Biological Products Co., Ltd. (Jiangsu, China). Glycerol (ACS reagent, anhydrous (99.5%)) was obtained from Yaohua Chemical Reagents Co., Ltd. (Tianjin, China). Sodium dodecyl sulfate (SDS), urea, sodium sulfide, ethanol, acetone, and sodium hydroxide of analytical reagent quality were bought from Tianli Chemical Reagents Ltd. (Tianjin, China). They were used without further purification in this study. Distilled water was employed for all solutions.

### 2.2. Extraction of Keratin from Yak Hair (USYK)

The USYK was extracted with urea and sodium sulfide according to our previously developed method [31]. The discarded yak hair was fully washed and degreased. The degreased yak hair was dried at 20 °C and cut into small pieces. Yak hairs (1 g) were added to the extraction buffer embodying 0.125 M SDS, 6 M urea, and 0.125 M sodium sulfide at a liquor ratio of 1:20 (weight of hair: volume of distilling water). The mixtures were intermittent agitated for 24 h at 60 °C. As a redox reagent, sodium sulfide can break the disulfide bonds between cysteine residues during extraction. As a result, the apparent viscosity (η_a_) of the solution decreased [32]. Then the extracted USYK solution was filtered with micro-filtration membranes (5 μm pore-size). After temperature reduction, the obtained solution was dialyzed (MWCO: 3500 Da) for 72 h at 20 °C and then lyophilized. The extracted USYK was seal preserved at 5 °C in the refrigerator. The weight-averaged (Mw) molecular weight of USYK was 7283 Da.

### 2.3. Preparation of the Isopeptide Bond Cross-Linked USYK-SPI Bioplastic Film

**Figure 1:**
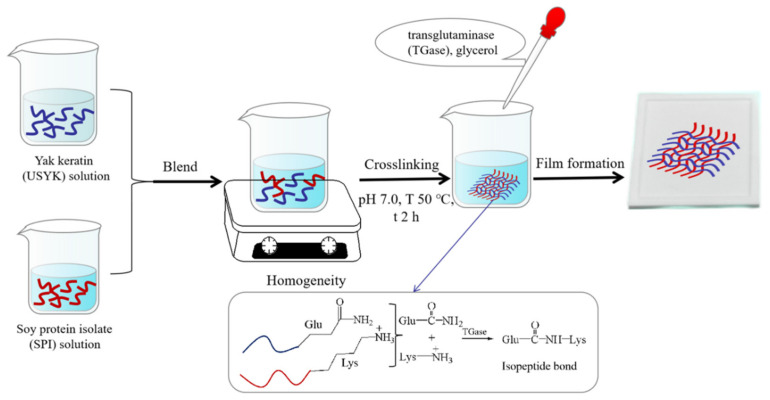
Schematic diagram of the preparation process for the USYK-SPI bioplastic film.

As shown in Figure 1, the USYK solutions (30 g/L) and the SPI solutions (30 g/L) were prepared separately. USYK (3 g) was added to 100 mL of distilled water and constantly stirring for 2 h at 20 °C. SPI (3 g) was added to 100 mL of distilled water and stirred constantly for 5 h at 70 °C. Then the USYK solution and the SPI solution were mixed with constant stirring for 1 h. The pH value of the mixing solution was adjusted to 7.0. TGase (15 U/g protein dry weight) was added to the mixing solution under constant stirring for 2 h at 50 °C. Then, the mixing solution was placed in a boiling water bath for 15 min. Subsequently, glycerol (10% protein dry weight) was added to the mixing solution as a plasticizer with constant stirring for 30 min when the mixing solution was cooled to 20 °C. The mixing solution was filtered and deflated. Finally, evenly mixed film-forming solutions (30 g) were poured into a polyTeflon plate (12 cm × 12 cm), dried at 20 °C, and uncovered. Before testing the film properties and structure, all USYK-SPI films were kept at a constant weight in a constant temperature and humidity box at 25 ± 0.5 °C with a relative humidity of 50 ± 5% RH for no less than 48 h, and stored in a plastic sealed bag at 20 °C.

### 2.4. X-ray Diffraction (XRD) Pattern

The crystal structure of the USYK-SPI bioplastic film was examined using a Rigaku X-ray Diffractometer (D8, Bruker Co. Saarbrücken, Germany) with CuKa radiation (X = 1.542 A) worked at 30 kV and 15 mA. The data obtained for the USYK-SPI bioplastic film were recorded within the 2θ range of 5 to 55° at a scanning speed of 2°/min.

### 2.5. Scanning Electron Microscopy (SEM)

The morphology of the cross-section of the USYK-SPI bioplastic film was analyzed using a scanning electron microscope (Q45, FEI Co., Hillsboro, NH, USA) at an accelerating voltage of 10 kV. The USYK-SPI bioplastic film was made brittle by liquid nitrogen, and then the brittle fracture of the film sample was mounted on an aluminum stub with double-sided adhesive tape and sprayed under a vacuum with gold. Then, the cross-sectional morphology of the USYK-SPI bioplastic film was observed.

### 2.6. The Thermal Behavior

The thermal behavior (TGA, DTG, and DSC) of the USYK-SPI bioplastic film was performed using a thermal analyzer instrument (STA449, Netzsch Ltd., Selb, Germany). The film sample was heated from 30 to 700 °C at a constant rate of 10 °C/min. Nitrogen atmosphere (25 mL/min) was employed as protective gas to prevent thermo-oxidative reactions during heating.

### 2.7. The Equilibrium Water Content

The USYK-SPI bioplastic films were produced with a constant weight using a dryer at a relative humidity of 50 ± 5% and the weight of the film samples was recorded as W_0_. Then, the film samples were dried in a drying box (105 °C) for 24 h, and the weight of the film samples was recorded as W_1_, while the dry weight (DW) of each film sample was calculated as:

(1) $$\mathrm{DW}\%=\frac{W_{0}-W_{1}}{W_{0}}\times 100$$

The equilibrium water content (EWC) of each film sample was calculated as:

EWC% = 100 − DW%(2)

All tests were implemented in sextuplicate.

### 2.8. Mechanical Properties

The thickness of the USYK-SPI bioplastic films was detected using a hand-held micrometer (MH-YDI, Golden Ocean Wanda Technologies Co. Ltd., Beijing, China), and five points of each film sample were randomly measured.

In accordance with ASTM/D638-91, the elongation at break (EAB) and the tensile strength (Ts) of the USYK-SPI bioplastic film were obtained using a tensile machine (Gotwill, AI-7000-NGD, Gotwill Co., Beijing, China). The film samples were cut into strips of 5 cm × 1 cm. The tensile rate was 5 mm/min, and the load cell had a voltage of 50 N. All USYK-SPI films were conditioned in a constant temperature and humidity box for 48 h at 50 ± 5% RH and 25 ± 0.5 °C before the measurement. All tests were implemented in sextuplicate.

### 2.9. Water Vapor Permeability (WVP)

In accordance with ASTM/E96-95, The water vapor transmission rate (WVTR) of the USYK-SPI bioplastic films was analyzed with a water vapor transmittance tester (W3/060, Jinan, China). The film samples (diameter of 74 mm) were placed into humidity chambers for 24 h at 25 °C. Three specimens were detected for each sample. The WVTR of each film sample was obtained from the linear slope of the weight gain. The water vapor permeability (WVP) of each film sample was calculated as:

(3) $$\mathrm{WVP}=\frac{\mathrm{WVTR}\times L}{\Delta P}$$

In formula 3, L is the thickness of the measured film, and ΔP is the difference in the water vapor partial pressure on the two sides of the measured film. All tests were implemented in triplicate.

### 2.10. Water Contact Angle (CA)

The water contact angle of the USYK-SPI bioplastic films was photographed at 20 °C using a surface contact angle instrument (OCA20, Dataphysics Co., Filderstadt, Germany) to estimate the hydrophobic character of each film sample. Ten specimens were used for each sample.

### 2.11. Light Transmittance Performance

The transmittances of the USYK-SPI bioplastic film were investigated using a UV-Vis spectrophotometer (Cary 5000, Agilent Technologies Co. Ltd., Palo Alto, CA, USA). The film samples were detected from 800 nm to 200 nm.

### 2.12. Statistical Analysis

SPSS Statistics 19.0 software (SPSS Inc., Chicago, IL, USA) was used for the analysis of variance. Duncan’s multiple-range method was used to test the differences among means. The confidence interval was set at 95% and the significance level was set at *p* ≤ 0.05.

## 3. Results and Discussion

### 3.1. Crystal Characteristics of the USYK-SPI Bioplastic Film

**Figure 2:**
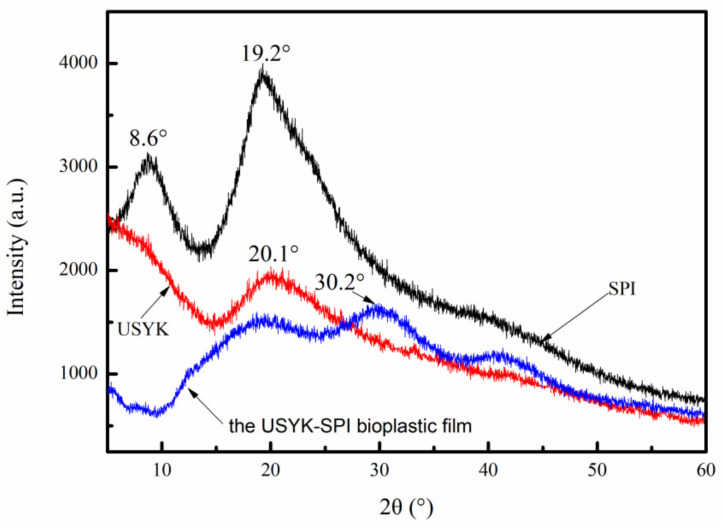
The X-ray diffraction (XRD) pattern of USYK, SPI, and the USYK-SPI bioplastic film (volume ratio: SPI/USYK of 3/1).

The formation process of the protein film plays a key role in determining its crystalline characteristics of the protein film [33]. The XRD patterns of the Gln-Lys isopeptide bond cross-linked USYK-SPI bioplastic film are shown in Figure 2. USYK and SPI are essentially amorphous. As shown in Figure 2, the XRD patterns of yak keratin, SPI, and the USYK-SPI bioplastic film have one typical diffraction peak at about 20°. This shows that the secondary structures of the yak keratin, SPI, and the USYK-SPI bioplastic film contain β-sheet structures separately [34]. It can be seen from Figure 2 that the USYK-SPI bioplastic film has weak crystalline characteristics. The highest diffraction peak occurs at 2θ = 30.2° in the XRD patterns of the USYK-SPI bioplastic film. This diffraction peak has a low d-spacing. The shift to a higher angle and decline in d-spacing show that the USYK-SPI bioplastic film has a long-range ordered structure [35]. This might be related to an ordered molecular arrangement caused by the low glycerol concentration in the USYK-SPI bioplastic film [36].

### 3.2. Cross-Sectional Micromorphology of the USYK-SPI Bioplastic Film

**Figure 3:**
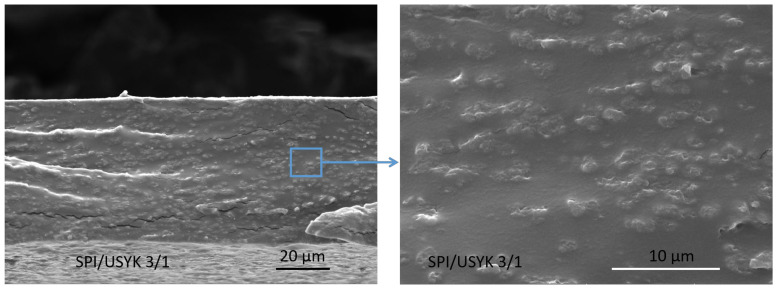
SEM photographs of the USYK-SPI bioplastic film (volume ratio: SPI/USYK of 3/1).

The typical microstructure of the USYK-SPI bioplastic film was observed by scanning electron microscopy (SEM), and the results are shown in Figure 3. As shown in Figure 3, although there were numerous near-spherical clumps with size of smaller than 10 µm were identified in the USYK-SPI bioplastic film, these clump sizes are less than the proper eye resolution. The USYK-SPI bioplastic films were found to have an omogeneous appearance [37]. The USYK-SPI bioplastic film displays a specific globular-shriveled structure. The formation of clumps suggests incomplete melting of the polymer components and the formation of aggregates that possibly reinforced polymer networks [37]. This further proves that SPI and USYK form a large number of protein complexes via Gln-Lys isopeptide bonds during TGase catalytic crosslinking. The different degrees of hydrophilicity in the protein complexes might lead to the formation of a micro-protrusion structure in the bioplastic film. The results show that TGase catalytic crosslinking enhances the compatibility between SPI and USYK. The addition of USYK to SPI can assist to prepare a rough microstructure of film through the self-polymerization of protein. The rough microstructure could provide favorable conditions for cell responses.

### 3.3. Apparent Properties of the USYK-SPI Bioplastic Films

The USYK-SPI bioplastic films were prepared at different volume ratios of SPI to USYK. All USYK-SPI films processed for this study exhibited a smooth surface and were flexible, highly transparent, and easy to strip from the mold. The effect of the water balance in protein film is the same as that of glycerol; it can effectively improve the flexibility of protein film. The thickness and equilibrium water content of the USYK-SPI film with different volume ratios of SPI to USYK were measured. The results are shown in Table 1. The thickness of USYK-SPI bioplastic films ranged from 0.08 mm to 0.10 mm. The thickness of the USYK-SPI bioplastic films changed slightly with different volume ratios of SPI to USYK. The thickness of the USYK-SPI bioplastic film reached its maximum value when the volume ratio of SPI to USYK was 5 during the formulation of bioplastic films. This indicates that the presence of large amounts of SPI has a filling effect on the keratin based composite films. Since the quality of the film-forming solution was strictly controlled during the film-forming process, the protein content in the film-forming solution was 30 g/L, and the volume ratio of SPI to USYK had no significant effect on the thickness of the bioplastic film. The equilibrium water content of the USYK-SPI bioplastic film was found to be around 19%. This result indicates the presence of strong hydrogen bonds between water molecules, USYK molecules, and SPI molecules.

### 3.4. Mechanical Properties of the USYK—SPI Bioplastic Films

**Figure 4:**
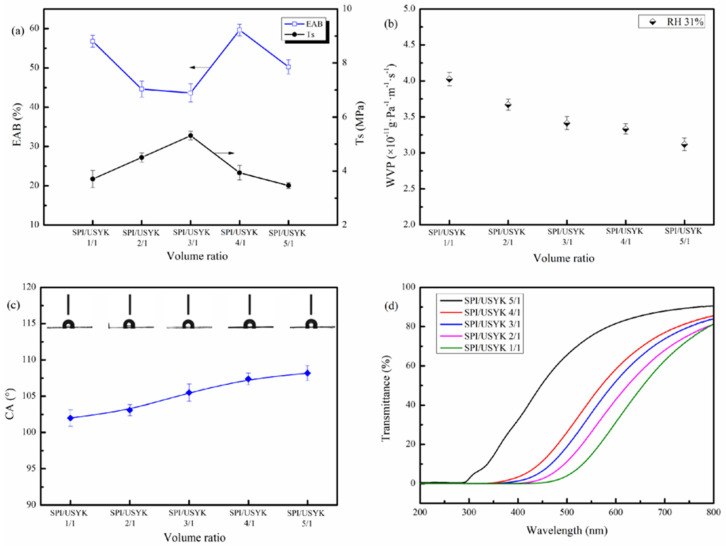
(**a**) The tensile strength (Ts) and elongation at break (EAB), (**b**) WVP, (**c**) contact angle (CA), and (**d**) UV-visible spectra of the USYK-SPI bioplastic films at different volume ratios (SPI/USYK of 5/1, 4/1, 3/1, 2/1 and 1/1).

The mechanical properties of the USYK-SPI bioplastic films were determined at different volume ratios of SPI to USYK by measuring the tensile strength (TS) and elongation at break (EAB). The results are shown in Figure 4a. As can be seen in Figure 4a, the Ts of USYK-SPI bioplastic films increased slightly while the EAB decreased as the content of SPI increased, suggesting improved rigidity of the films. The added SPI possibly acted as a reinforcement, increasing the strength of the material, while the inhomogeneity of the keratin-based film matrices reduced the elongation [38,39]. The TS of USYK-SPI bioplastic film reached maximal strength when the volume ratio of SPI to USYK was 3 during the formulation of bioplastic films. When TGase was used as a crosslinker, a large number of new Gln-Lys isopeptide bonds would be produced between amine and glutamate residues [40]. Gln-Lys isopeptide bond crosslinking between the USYK and the SPI proteins prompted conformational changes in the two protein molecules and restricted the mobility of USYK and the SPI polypeptide chains and increased the rigidity of the USYK-SPI polymers [41]. The self-aggregation of USYK and the SPI polypeptide chains could have conferred a compact film-forming structure and made the USYK-SPI bioplastic film stronger with less deformation. Furthermore, the results show that the crosslinking interactions between the amino and carboxyl groups of SPI and USYK might promote the mechanical strength of the bioplastic films. However, when the volume ratio of SPI to USYK was greater than 3, with a further increased in the content of SPI, the strength of the USYK-SPI bioplastic film reduced, while the elongation increased. This might have been due to the high amounts of SPI possibly enhancing formation of continuous SPI networks, forming co-continuous polymer matrices, which reduced the rigidity of the films, leading to a reduced Ts and improved EAB [42]. As can be seen from the EAB data of the USYK-SPI bioplastic films, the USYK-SPI bioplastic films had great extendable capacity. The results suggest that SPI with a spherical structure has more wrinkles, which could provide a certain buffer for stretching [6]. This is consistent with the SEM data (Figure 3).

### 3.5. Water Vapor Permeability of the USYK-SPI Bioplastic Films

The water vapor permeability (WVP) is an important indicator that mediates the moisture evaporation of bioplastic film. Satisfactory water vapor permeability is desired to effectively prevent the rapid evaporation of water. Molecular ordering in the film structure is a key factor in determining the WVP of the film. As shown in Figure 4b, the introduction of SPI led to a certain decrease in the permeability of bioplastic film. This was probably because the molecular conformation of SPI and USYK changed during Gln-Lys isopeptide bond crosslinking, and the inherent intermolecular forces in the two proteins were weakened. With the increase in Gln-Lys isopeptide bonds between the protein molecules, the polypeptide chains of USYK and SPI became intertwined, which further increased the curvature of polypeptide chains in the bioplastic film. At the same time, hydrophilic proteins are consumed during crosslinking [34]. The enhanced dispersion and formation of co-continuous protein matrices possibly produced denser networks, limiting the diffusion of water vapor and volatile compounds in the USYK-SPI bioplastic films [43]. Furthermore, it is possible that SPI contains more hydrophobic amino acid residues than USYK, contributing to the increased hydrophobicity of the matrices and decreasing the diffusion of water vapor [44], consequently reducing the diffusion of water molecules and making it difficult to penetrate the USYK-SPI bioplastic film. The results show that the USYK-SPI bioplastic film had an appropriate level of water vapour permeability.

### 3.6. Surface Hydrophobicity of the USYK-SPI Bioplastic Films

Contact angles (CA) can be used to evaluate the surface hydrophobicity of the biofilms. The contact angles (CA) of the USYK-SPI bioplastic films are shown in Figure 4c. As can be seen in Figure 4c, the incorporated SPI possibly improved the surface roughness, which subsequently improved the contact angle values [37]. Moreover, the introduction of SPI prompted the relocation of hydrophobic groups on USYK molecules, which endowed the USYK-SPI bioplastic film with stronger surface hydrophobicity. Some hydrophilic functional groups (amino groups and carboxyl groups) might be consumed during Gln-Lys isopeptide bond cross-linking. In order to reduce the energy required, the more hydrophilic functional groups were hidden, and the more hydrophobic groups were exposed on the surface of USYK-SPI bioplastic film. As a result, the surface hydrophobicity of the USYK-SPI bioplastic film was enhanced through the introduction of SPI, and the transport and adsorption speed of water molecules in the bioplastic film became slower.

### 3.7. UV Barrier Properties of the USYK-SPI Bioplastic Films

The UV-visible spectra of USYK-SPI bioplastic films with different volume ratios of SPI to USYK are exhibited in Figure 4d. As can be seen from Figure 4d, the USYK-SPI bioplastic films had almost zero transmission value in a wavelength range of 200–300 nm, indicating excellent UV barrier properties. This is mainly due to the SPI and USYK molecules possessing aromatic amino residuals (tyrosine, phenylalanine, tryptophan), which can absorb ultraviolet radiation, giving the USYK-SPI bioplastic films excellent UV barrier properties. The TGase crosslinking had no side effects on the UV barrier properties of the bioplastic film. Our research group also found that the collagen-SPI blend film had UV barrier properties [45]. Meanwhile, the visible light transmittance of the USYK-SPI bioplastic film increased with the increase of the volume ratios of SPI to USYK. This might be related to the color of the extracted USYK solution and the yellowing of the bioplastic during TGase catalytic crosslinking. It was inferred that there was an obvious phase interface occurs in USYK-SPI bioplastic film and that visible light is reflected and scattered at the phase interface between the dispersed phase and continuous phase.

### 3.8. Thermal Properties of the USYK-SPI Bioplastic Film

**Figure 5:**
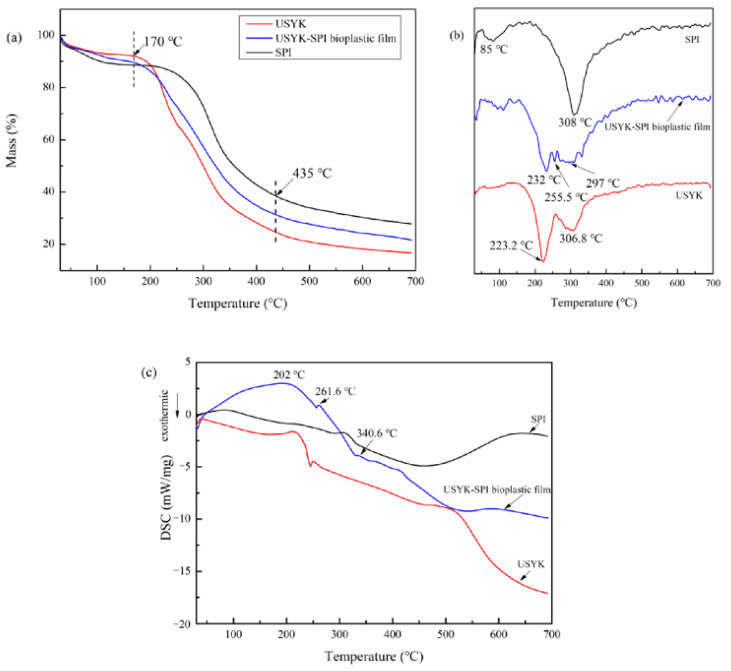
TG (**a**), DTG (**b**), and DSC (**c**) curves of USYK, SPI, and the USYK-SPI bioplastic film (volume ratio: SPI/USYK of 3/1).

The TGA curves of yak keratin, SPI, and USYK-SPI bioplastic film are displayed in Figure 5a. As can be seen in Figure 5a, the thermal weightlessness of yak keratin, SPI, and the USYK-SPI bioplastic film sample showed three distinct stages as the temperature increased. The quality loss in the first stage (<170 °C) was related to the evaporation of absorbed water and crystal water. The main quality loss was concentrated in the second stage (170–435 °C). The quality loss of the USYK-SPI bioplastic film was about 60% at this stage. A slippery slope appeared on the TGA curves. This suggests that the Gln-Lys isopeptide bonds and peptide bonds of the SPI molecules and the USYK molecules formed during thermal decomposition. The pyrolysis of protein molecules was mainly concentrated in this stage [46]. The quality loss in the third stage (>435 °C) was related to the thermal decomposition of residual carbons.

It is well known that the heating process could disrupt the crosslinking structure of the USYK-SPI bioplastic film and further lead to the helix-coil transition of protein molecules. The DTG curves of yak keratin, SPI, and USYK-SPI bioplastic film are presented in Figure 5b. The thermal decomposition peaks relevant to USYK, SPI, and USYK-SPI bioplastic film degradation are presented on their respective DTG curves. Compared with the quality loss temperature of a single USYK, the largest quality loss temperature of the USYK-SPI bioplastic film was increased to some extent. Thermal degradation of the main polymer backbone in USYK-SPI bioplastic film began at 232 °C. The results indicate that a large number of Gln-Lys isopeptide bonds and hydrophobic interactions were produced between SPI and USYK following to TGase treatment. The formation of Gln-Lys isopeptide bonds and hydrophobic interactions led to the production of a stable crosslinking structure in the USYK-SPI bioplastic film. Thus, a higher temperature is required to break chemical bonds and unfold the protein molecules in the USYK-SPI bioplastic film [6]. The USYK-SPI bioplastic film showed an anticipated improvement in thermal stability with the addition SPI.

The DSC curves of yak keratin, SPI, and USYK-SPI bioplastic film in the temperature range of 30–700 °C are shown separately in Figure 5c. The DSC curves of the USYK-SPI bioplastic film display three distinct peaks. The large endothermic peak at 202 °C from a new crystalline phase indicates α-crystallites denaturation of the USYK-SPI bioplastic film. The second endothermic peak at 261.6 °C indicates the β-sheet denaturation of the USYK-SPI bioplastic film. The two endothermic peaks at 202 °C and 261.6 °C coincide with the crystallinity in the USYK. The third endothermic peak at 340.6 °C indicates complete degradation of the USYK-SPI bioplastic film and glycerol.

## 4. Conclusions

To achieve agro-industrial waste treatment and introduce resource recycling into the yak-related industry, an eco-friendly Gln-Lys isopeptide bond cross-linked bioplastic film was prepared in our study by using USYK from yak hair and SPI from soybean meal. TGase, as a crosslinker, and glycerol, as a plasticizer, were employed during USYK-SPI bioplastic film production. Since USYK and SPI have certain synergistic effects, the structure and properties of the USYK-SPI bioplastic film were optimized through TGase enzymatic catalytic crosslinking technology. The results reveal that the thermal and mechanical behaviors of USYK-SPI bioplastic film were improved. Furthermore, the UV barrier, water permeability, and surface hydrophobicity properties of the USYK-SPI bioplastic film were enhanced. This novel protein bioplastic film could potentially be used to exploit packaging materials, biomedical materials, eco-friendly wearable electronics, and humidity sensors, etc. The high-value conversion of discarded yak hair and soybean meal into new biological functional protein film is in line with the strategic direction of sustainable development.

## Figures and Tables

**Table 1:** Thickness and EWC of the USYK-SPI bioplastic films.

Volume Ratios (SPI/USYK)	5/1	4/1	3/1	2/1	1/1
Thickness (mm)	0.1 ± 0.006	0.08 ± 0.007	0.09 ± 0.009	0.09 ± 0.006	0.08 ± 0.009
EWC (%)	19.24 ± 0.23	19.54 ± 0.15	19.08 ± 0.16	19.52 ± 0.11	19.82 ± 0.33

## Data Availability

The data presented in this study are available on request from the corresponding author.
